# Use of the Appendix as Ureteral Substitute in a Patient with a Single Kidney Affected by Relapsing Upper Urinary Tract Carcinoma

**DOI:** 10.1100/tsw.2005.36

**Published:** 2005-04-05

**Authors:** Alessandro Antonelli, Danilo Zani, Paolo Dotti, Luigi Tralce, Claudio Simeone, Sergio C. Cunico

**Affiliations:** Department of Urology, University of Brescia, c/o Divisione di Urologia – Piazzale Spedali Civili no.1, 25127 Brescia, Italy

**Keywords:** appendix, upper urinary tract, transitional cell carcinoma, ureteral cancer, ureteral replacement

## Abstract

The treatment of upper urinary tract transitional cell carcinoma (UT-TCC) in single-kidney patients requires the radical removal of cancer, but also, when feasible, the preservation of the continuity of the urinary tract by various surgical techniques. In case of wide resections during ureteral surgery, a ureteral replacement could be advocated. In the literature, the cecal appendix has rarely been used as a ureteral substitute, moreover in benign pathological conditions, showing encouraging early results. The positive functional and oncological outcomes obtained after a lengthy follow-up in a single-kidney patient with UT-TCC treated by ureteral resection and appendix interposition confirm the viability of this surgical option.

## INTRODUCTION

Although the cecal appendix has long been used in urologic surgery as a continent catheterization way for the bladder or urinary reservoirs, literature reports on its use as a ureteral substitute are rare, especially with relation to the sparing treatment of upper urinary tract transitional cell carcinoma (UT-TCC). We report our experience with a single case out of 237 submitted to surgery for UT-TCC at our department.

## CASE REPORT

In July 1998, N.G., male, 77, diabetic, hypertensive, underwent left nephroureterectomy, right terminal ureterectomy with ureterocystoneostomy on bladder psoas hitching, and TURBT because of a pT1G2 synchronous bilateral transitional carcinoma affecting the left renal pelvis and the right pelvic ureter and a T1G2 bladder transitional carcinoma.

After 12 months, following an episode of macrohematuria, a filling defect of the lumbar-pelvic ureter, which was compatible with a relapsing carcinoma, was shown by urography and ascending ureteropyelography (Fig. 1) while cystoscopy did not reveal any bladder relapses.

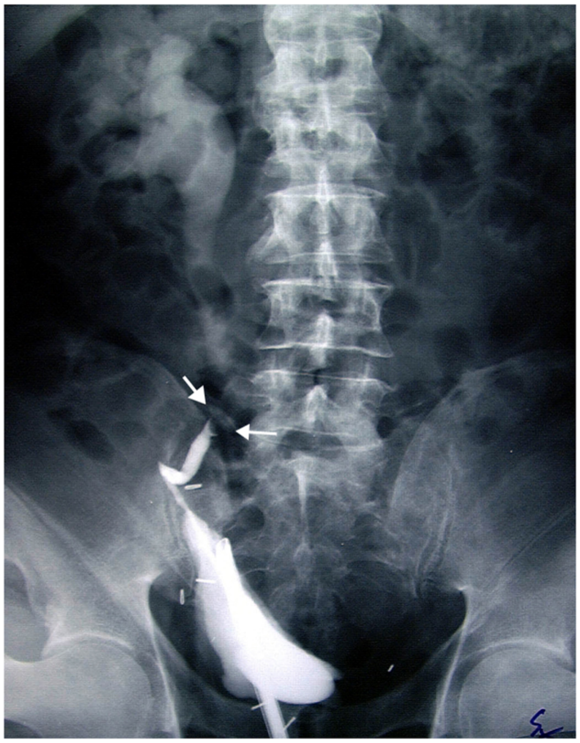

**FIGURE 1.** Ascending ureteropielography highlighting a ureteral filling defect (note the delayed clearance of contrast medium of the previous urography).

During a second operation, after a wide segmentary ureterectomy with a disease-free proximal ureteral margin, the availability of a 10-cm long appendix with a normal macroscopic aspect made us decide to use it. After opening the appendix tip, its patency was checked by means of a 12-Ch catheter. With the help of a ureteral tutor MJ 8 Ch, the appendix was interposed between the ureter and the bladder antiperistaltically, so as to preserve its physiological orientation (base at the top and tail at the bottom) and to avoid the risk of rotation on the appendicular meso (Fig. 2). Ureteroappendicular anastomosis was performed by spatulating the ureter stump, while ureterovesical anastomosis was performed directly extravesically.

Neither intra- nor perioperative complications occurred. Histological examination revealed a pT1G3 transitional cell papillary carcinoma of the ureter. After a temporary worsening, renal function tests gave results similar to preoperative levels, with creatininemia at approximately 1.6 mg/dl. Following a radiological examination 10 days after surgery showing no signs of leakage, the MJ ureteral stent was replaced with a DJ, which was kept in place for 30 days and then definitely removed. Renal functional tests, urinary cytology, cystoscopy, and urography (Fig. 3) are normal at 60 months from surgery.

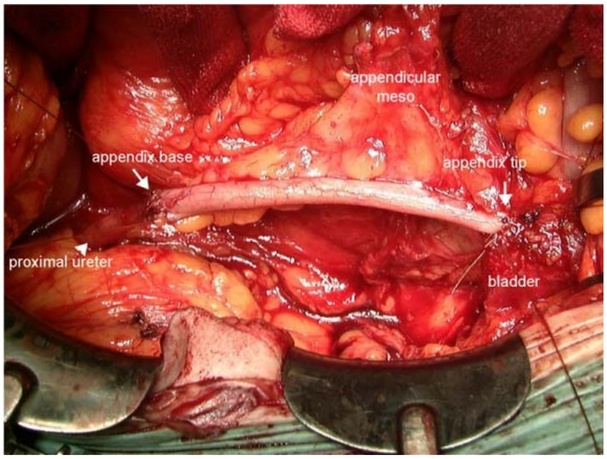

**FIGURE 2.** Antiperistaltic interposition of the appendix.

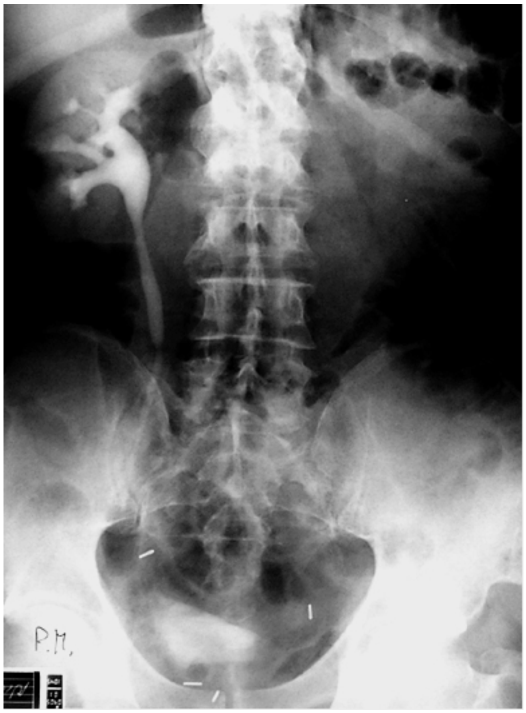

**FIGURE 3.** Urography after 60 months.

## DISCUSSION

Several pathological conditions affecting or secondarily involving the excretory tract may require large ureteral resections. The resulting gap can be corrected using different surgical techniques (psoas hitching, Casati-Boari bladder-flap, renal reverse hitching, renal autotransplantation). However, when the loss of ureteral tract is significant, full or partial replacement or the ureter is required[1].

The cecal appendix, which has long been used in urologic surgery as an intermittent catheterization stoma for the bladder or urinary reservoirs (Mitrofanoff principle)[2], has been used to replace the right and, more rarely, the left ureter in various pathological conditions[3,4,5,6,7,8]. Compared to the ileum and colic segments, it provides several advantages: suitable caliber/length ratio, little tendency to dilation over time, caliber and structure comparable with ureteral ones, exclusive vascularization, reduced mucosa surface and little reabsorption of urinary electrolytes, and easy removal with no need for intestinal anastomosis. In spite of all these advantages, very few reports can be found in the literature, with only two of them related to patients affected by UT-TCC[9,10], due to the high prevalence of appendicectomized patients and the incidence of anomalous or pathological appendixes (up to 90% on appendectomies performed with no clinical evidence of appendix pathology), especially among elderly patients[11]. Some authors suggest interposition of the appendix isoperistaltically to favor the urine flow with the peristalsis of the appendix[10], but in our experience, reverse (antiperistaltic) interposition had no immediate or late consequences on urine flow from the upper excretory tract. At the same time, this way of interposition reduces the risks of ischemia secondary to a kinking of the appendicular meso during its rotation when the appendix is isoperistaltically interposed. In our opinion, the dehiscence of the ureteroappendicular anastomosis after isoperistaltic interposition reported by some authors[3,10] could be a consequence of the ischemia of the appendicular tail, less vascularized also in physiological conditions. In our case, the length of the ureteral gap and the previous bladder psoas hitching prevented us from performing ureterovesical anastomosis using antireflux technique, but this fact had no clinical relevance over time. We believe that lower absorption of urinary electrolytes by the appendix compared to the ileal segment contributed to the preservation of an only slightly compromised renal function in this patient. Finally, it is remarkable that 5 years after surgery, the patient has had no relapses of the transitional carcinoma, neither in the upper nor in the lower excretory tract.

## CONCLUSION

Our experience confirms that the appendix, when available and disease free, is a suitable ureter substitute. Its use is an option worth considering in the sparing surgery of the upper excretory tract carcinoma.
